# Right-Side Endocarditis: A Typical Presentation in an Atypical Patient

**DOI:** 10.7759/cureus.18897

**Published:** 2021-10-19

**Authors:** João P Pais, Marta Sousa, Rita Mota, Ana R Cambão, Ana Nascimento

**Affiliations:** 1 Internal Medicine, Unidade Local de Saúde do Alto Minho (ULSAM) - Hospital de Santa Lúzia, Viana do Castelo, PRT

**Keywords:** septic pulmonary embolism, streptococcus mitis, pulmonary embolism, heart failure, tricuspid valve endocarditis

## Abstract

Right-side endocarditis (RSE) is a well-defined clinical entity, rarer than left-side endocarditis. Known risk factors include intravenous drug use or the presence of medical devices. The most frequently affected valve is the tricuspid valve. In most cases, medical treatment is enough. Surgical treatment is reserved for failed medical therapy or in the presence of large vegetations. Although there is a high recurrence rate in intravenous drug users (IDU), RSE has a generally good prognosis. We present the case of a 70-year-old male with no known previous diseases other than alcohol abuse. He was admitted with fever, cough, hemoptysis and a weight loss of 8 kg in two months. Chest X-ray revealed two images of condensation, one in the right pulmonary base and another in the superior right lobe. A computerized tomography of the thorax revealed a subsegmental pulmonary embolism. The patient refused hospitalization and was discharged medicated with levofloxacin and apixaban. In ambulatory, there was a decrease in size of the lesions but with a new lesion in the right hemithorax. Two months after the first episode, the patient is admitted with the same symptoms. The transthoracic echocardiogram showed a 20cm vegetation in the tricuspid valve. He was admitted to the hospital and received treatment with penicillin and gentamicin after isolation of *Streptococcus mitis* in the blood cultures. Surgical treatment was needed after a weak response to antibiotics, with a good evolution.

## Introduction

Right-side endocarditis (RSE) is an uncommon condition, comprising only 5-10% of all cases of infectious endocarditis [1-4]. The diagnosis of RSE is usually associated with the presence of risk factors, like intravenous drug usage (IDU), patients with intravenous devices, such as pacemakers and central venous catheters, and with congenital heart disease [2-4]. The main risk factor for developing RSE is IDU, with 86% of total cases of infective endocarditis in this population being right-sided, mainly affecting the tricuspid valve [1,4,5]. In most cases, one of these risk factors is identified when the diagnosis of RSE is made. In some cases, none of these risk factors is identified and these patients are usually older and with multiple comorbidities leading to a worse prognosis compared to IDU patients [6].

RSE typically affects the tricuspid valve, with isolated involvement of pulmonary valve being a rare condition [1,4]. The usual presentation is similar to a respiratory infection. Presenting symptoms include persistent fever and respiratory symptoms, like shortness of breath and productive cough [2,3]. Because of the likeness to a respiratory infection, diagnosis of RSE is usually delayed, thus a high level of suspicion is needed to make the proper diagnosis in due time [1].

*Staphylococcus aureus* is the major microbiological cause of RSE, which is associated with the usage of intravenous drugs and cardiac and intravenous devices as the main risk factor for developing the disease [2,4]. As in most infections, an increased incidence of methicillin-resistant microorganisms is one of the main concerns regarding RSE [7].

Usually, antibiotic treatment alone is enough to treat RSE. In some cases, surgical treatment may be necessary, mainly in the presence of larger vegetations, recurrent septic emboli, or failure of medical therapy alone [7].

## Case presentation

**Figure 1:**
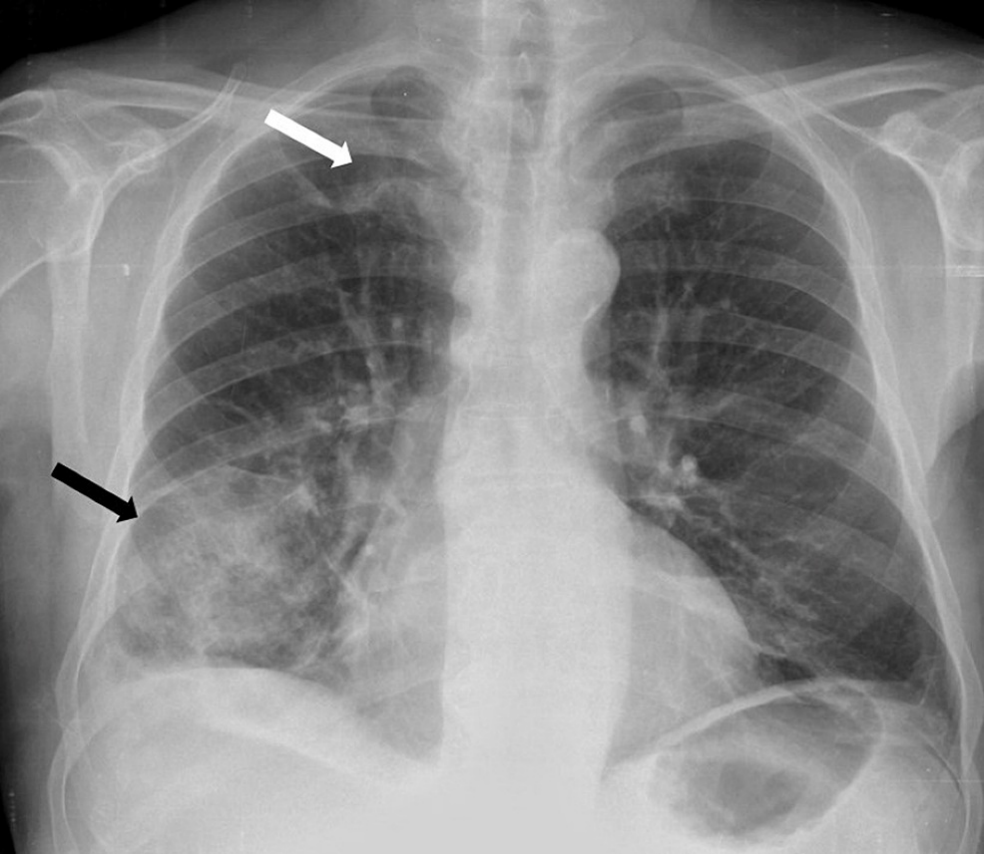
Chest X-ray obtained in the first observation, revealing two images of condensation, in the right pulmonary base (black arrow) and in the superior right lobe (white arrow).

**Figure 2:**
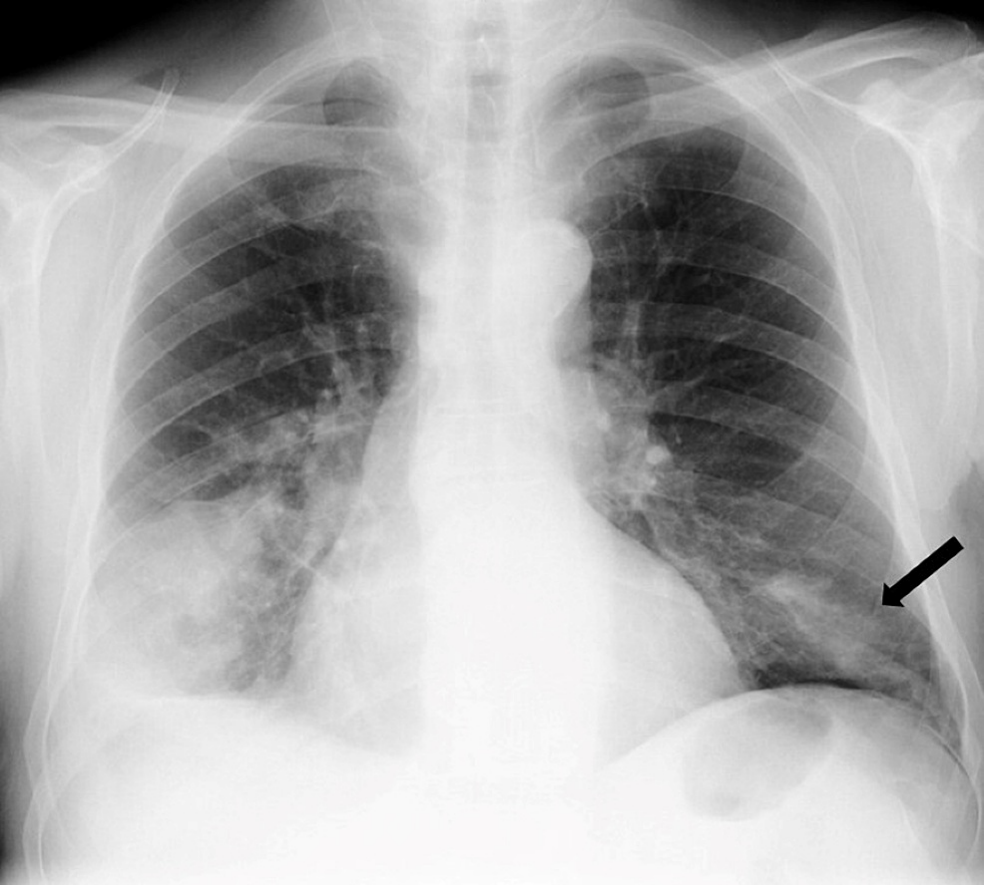
Chest X-ray obtained one week after the first observation in the emergency department, with a new condensation in the left inferior pulmonary lobe (black arrow).

We present the case of a 70-year-old male without previous known diseases except from alcohol abuse (about 100g of alcohol per day), with no usual medication. He denied prior or current use of intravenous drugs. He presented in the emergency department (ED) with a two-month history of chest pain in the left hemithorax, which was aggravated with respiratory movements. He also had complaints of general weakness and loss of weight of 8 kg. The chest X-ray revealed two images of condensation, one in the right pulmonary base and another in the superior right lobe (Figure 1). The computerized tomography (CT) of the thorax revealed a subsegmental pulmonary embolism and two irregular pulmonary nodules. The patient refused hospitalization and went home medicated with levofloxacin and apixaban. He was referred to outpatient setting for further investigation, namely bronchofibroscopy. One week after the first ED episode, a new chest X-ray was done before the bronchofibroscopy, with a new condensation in the left inferior pulmonary lobe (Figure 2). The bronchofibroscopy did not show any structural endobronchic abnormality and the bronchoalveolar lavage had no microbiologic or cytologic alterations.

**Figure 3:**
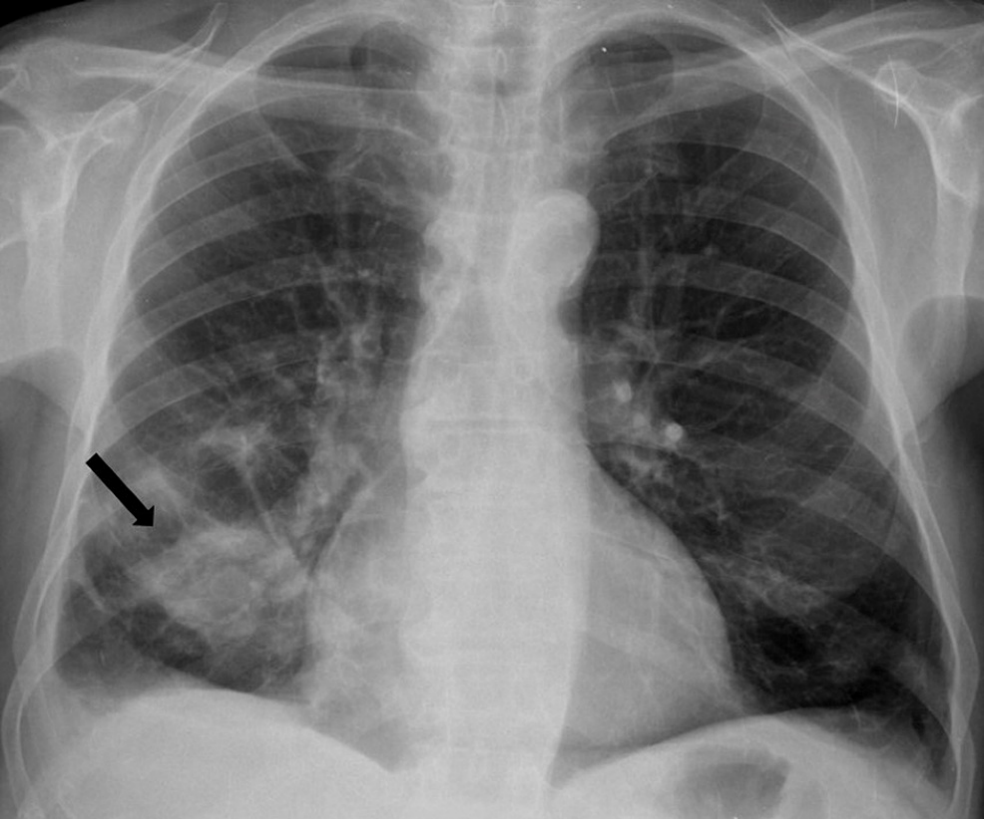
Chest X-ray obtained three weeks after the first observation, revealing a new condensation in the inferior lobe of the right lung (black arrow).

Two weeks after the bronchofibroscopy, the patient returned to the ED. He maintained chest pain, with the same characteristics already described. He also referred complaints of productive cough, with mucopurulent and sometimes hemoptoic sputum and fever, with a maximum temperature of 38.5ºC, with a daily peak. These new symptoms appeared after the bronchofibroscopy. At our observation, he was pale, hypotensive (108/70 mmHg), had diminished respiratory sounds in the inferior half of both lungs and had bilateral expiratory crackles. The cardiac auscultation was normal, with no heart murmurs identified. A new chest X-ray was made, with a condensation in the inferior lobe of the right lung (Figure 3). In the blood tests, we identified normocytic and normochromic anemia, leucocytosis, with neutrophilic predominance, a C-reactive protein of 10.12 mg/dL, and an erythrocytic sedimentation rate of 63 mm. The patient was hospitalized for treatment and further investigation of the lung lesions. Blood for microbiologic cultures was obtained, and antibiotic therapy with a carbapenem was initiated.

**Figure 4:**
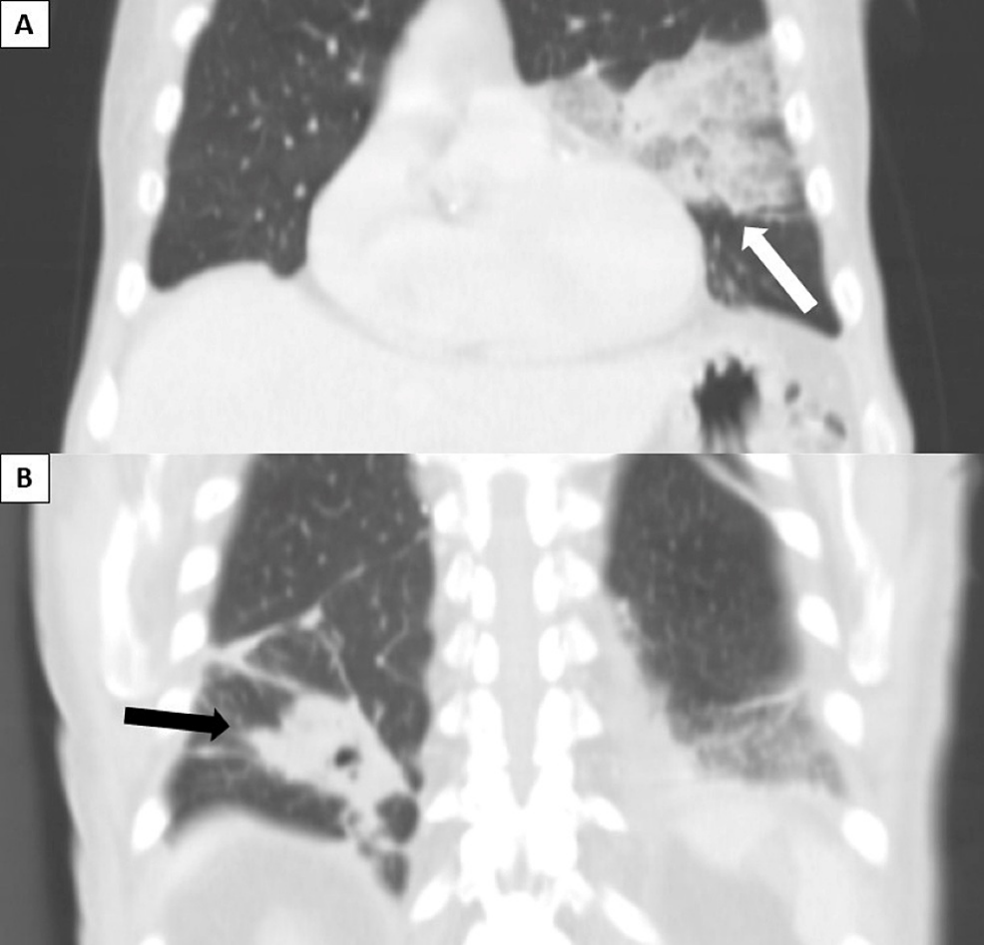
Computerized tomography of the thorax obtained after hospitalization of the patient, revealing two areas of consolidations, one in the lingula (panel A, white arrow) and another in the inferior lobe of the right lung (panel B, black arrow).

**Figure 5:**
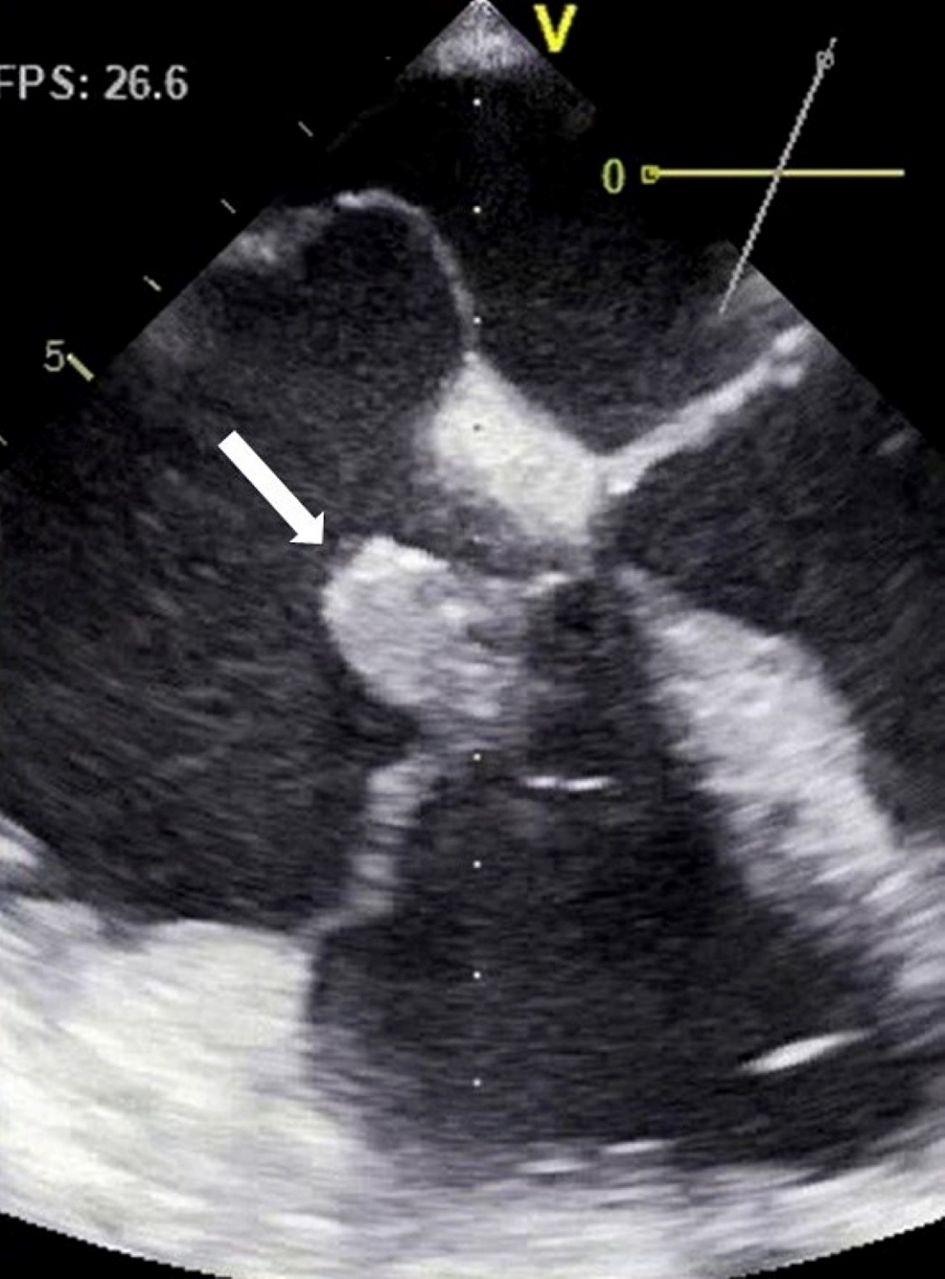
Transthoracic echocardiogram obtained during hospitalization, revealing a mobile tricuspid vegetation (white arrow), with dimensions of 22x18 mm.

The thoracic CT scan was repeated during hospitalization, revealing two areas of consolidation, one in the lingula and another in the inferior lobe of the right lung (Figure 4), both images suggestive of infectious etiology. A transthoracic echocardiogram was made revealing a vegetation on the tricuspid valve (Figure 5). Transesophagic echocardiogram confirmed the presence of the vegetation, the blood cultures were positive, with the identification of *Streptococcus mitis* in all the collected samples, and the diagnosis of right-side endocarditis was made. Antibiotic treatment was changed to penicillin and gentamicin, with significant clinical and analytical improvement. New blood cultures were obtained after three days of treatment, which came negative. The transthoracic echocardiogram was repeated after 14 days of treatment, which showed the same vegetation with overlapping dimensions. This was considered as an insufficient response to medical treatment, and the patient underwent surgical substitution of the tricuspid valve with a biological prosthetic valve.

After surgery, antibiotic treatment was continued until completing four weeks of treatment, with good clinical and analytical improvement.

## Discussion

RSE is rarer than left-side endocarditis, corresponding to 5-10% of all cases of endocarditis [1-4]. Although intravenous drugs use is strongly associated with RSE, other risk factors have been identified, such as implantation of medical devices, uncorrected congenital heart diseases, and immunosuppression [4,7]. The tricuspid valve is affected in about 90% of all cases of RSE, while isolated pulmonary valve involvement is only seen in 5% of cases [1,3]. *Staphylococcus aureus* is the most frequent microorganism responsible for RSE, being responsible for more than half the cases of RSE [7]. Viridans streptococci, like *S. mitis*, are more commonly isolated in cases of RSE in non-intravenous drug users, ranging from 10% to almost 30% [2,8]. The clinical presentation of RSE commonly resembles a respiratory infection, with patients usually complaining of fever, dyspnoea, cough, and pleuritic chest pain [1,3], making a possible misdiagnosis very likely [9]. These cases are frequently treated as respiratory infections. As transitory improvement can be seen with antibiotic treatment, many cases of RSE remain undetected, or the diagnosis is delayed [9]. The so-called “tricuspid syndrome” comprised of respiratory symptoms, normocytic anemia and microscopic haematuria should raise suspicion for the presence of RSE [1,3]. Treatment should be initiated as soon as possible to avoid high morbimortality. Usually, medical treatment is enough to cure patients with RSE [4,9]. However, about 25% of the patients will need surgical treatment, although optimized antibiotic therapy [1,4]. According to current guidelines, surgery should be considered when right heart failure develops secondary to severe tricuspid regurgitation with poor response to diuretic therapy, when bacteriaemia is present for at least seven days despite adequate antimicrobial therapy, and when tricuspid valve vegetations are over 20 mm in diameter and are associated to recurrent pulmonary emboli [10]. In the described case, the non-resolving vegetation with over 2 cm diameter although adequate antibiotic therapy was a clear indication for a surgical approach. The prognosis of RSE is usually good, probably due to minimal hemodynamic instability caused by right-side valvular dysfunction and the septic emboli [1] with mortality rates usually below 10%.

## Conclusions

RSE is a rare pathological entity with a clinical presentation similar to a respiratory infection. Although more frequent in IDU or patients with cardiac devices or central catheterization, it is observed in patients without any of these risk factors in some rarer cases. Therefore, a high level of suspicion is needed to identify and treat this disease correctly.
